# Single Nucleotide Polymorphisms in MCP-1 and Its Receptor Are Associated with the Risk of Age Related Macular Degeneration

**DOI:** 10.1371/journal.pone.0049905

**Published:** 2012-11-21

**Authors:** Akshay Anand, Neel Kamal Sharma, Amod Gupta, Sudesh Prabhakar, Suresh Kumar Sharma, Ramandeep Singh, Pawan Kumar Gupta

**Affiliations:** 1 Department of Neurology, Post Graduate Institute of Medical Education and Research (PGIMER), Chandigarh, India; 2 Department of Ophthalmology, Post Graduate Institute of Medical Education and Research (PGIMER), Chandigarh, India; 3 Department of Statistics, Panjab University, Chandigarh, India; Tor Vergata University of Rome, Italy

## Abstract

**Background:**

Age-related macular degeneration (AMD) is the leading cause of blindness in the elderly population. We have shown previously that mice deficient in monocyte chemoattractant protein-1 (MCP1/CCL2) or its receptor (CCR2) develop the features of AMD in senescent mice, however, the human genetic evidence so far is contradictory. We hypothesized that any dysfunction in the CCL2 and its receptor result could be the contributing factor in pathogenesis of AMD.

**Methods and Findings:**

133 AMD patients and 80 healthy controls were enrolled for this study. Single neucleotid Polymorphism for CCL2 and CCR2 was analyzed by real time PCR. CCL2 levels were determined by enzyme-linked immunosorbent assay (ELISA) after normalization to total serum protein and percentage (%) of CCR2 expressing peripheral blood mononuclear cells (PBMCs) was evaluated using Flow Cytometry. The genotype and allele frequency for both CCL2 and CCR2 was found to be significantly different between AMD and normal controls. The CCL2 ELISA levels were significantly higher in AMD patients and flow Cytometry analysis revealed significantly reduced CCR2 expressing PBMCs in AMD patients as compared to normal controls.

**Conclusions:**

We analyzed the association between single neucleotide polymorphisms (SNPs) of CCL2 (rs4586) and CCR2 (rs1799865) with their respective protein levels. Our results revealed that individuals possessing both SNPs are at a higher risk of development of AMD.

## Introduction

Age related macular degeneration is the leading cause of irreversible blindness in the elderly population [1], [2]. AMD is of two types: early and late. In the early stage of disease there is presence of drusen with pigmented and hyperpigmented area. After the disease progresses with time, it enters into the second stage i.e. the late stage. The early one is dry or atrophic AMD, which is marked by geographic atrophy or sharply demarcated area of depigmentation caused by waste by products of the retinal pigment epithelium (RPE) and photoreceptors. The late stage of disease is called wet AMD as it occurs because of the growth of new blood vessels under the RPE and neurosensory retina, which results in subretinal bleeding and subsequent scar formation [3]. The complete mechanism of age-related macular degeneration (AMD) is not well understood. In recent years, there has been increasing evidence of an inflammatory component in AMD. It has been found to be associated with polymorphism of complement factor H (CFH) [1], [2], a polymorphism which leads to an overactivation of the complement system [3], emphasizing the importance of inflammatory mediators in AMD.

During past few years, certain studies have also focused on the role of chemokines in the progression of AMD. Although the mechanisms underlying the regulation of these cytokines in the eye of patients with AMD remain unclear, chemokines like MCP-1, while acting in concert with receptor CCR2, promote recruitment of macrophages [4]. We hypothesized that any dysfunction in the CCL2 and CCR2 results in impaired macrophage recruitment and debris formation under the retinal pigment epithelium (RPE) contributions to AMD. CCL2 gene is located on chromosome 17q11.2 while CCR2 is located on chromosome 3p21.31.We previously described the spontaneous development of CNV in senescent mice deficient in CCL2 or its CCR2 receptor [4]. Besides, many recent reports have suggested that inflammation is the major cellular process that plays main role in the pathogenesis of AMD [5] and its development to CNV [6]. Some RPE cells play essential role in the maintenance of outer retina by secreting cytokines including CCL2 [7], which have been suggested to be implicated in the pathogenesis of AMD [8]. RPE cells can secrete CCL2 in the direction of choroidal blood vessels during inflammatory reaction suggesting that RPE cells might promote macrophage recruitment to the choroid from circulating monocytes.

There are a few studies which have examined SNPs of the chemokine system with AMD susceptibility but did not find any evidence of association between CCL2, CCR2 and AMD [9], [10]. The absence of any such genetic association studies between CCL2 or CCR2 and AMD from Indian patients prompted us to explore the role of these chemokines in these patients. We analyzed whether single nucleotide polymorphism (SNP) variants in the CCL2 or CCR2 loci independently or in combination are associated with AMD as different ethnic groups may exhibit a varying spectrum of SNPs.

## Methods

### Study Population

The study was approved by the Ethics Committee of Post-Graduate Institute of Medical Education and Research, Chandigarh, India vide letter No Micro/10/1411. The written informed consent was obtained from participants for the study, as well as for the publication of the data obtained after retrieval of medical records, besides use of blood and DNA for AMD related research project. All the patients were scored at the base line. Individuals with AMD in at least one eye were recruited between 2008 to 2011 from Advanced Eye Centre, Post-Graduate Institute of Medical Education and Research, Chandigarh (PGIMER), India.

We included 213 case-control samples consisting of 133 AMD patients from Eye Centre, PGIMER, with 80 genetically unrelated healthy controls as per inclusion and exclusion criteria described below. Out of 133 AMD and 80 control samples, about nine samples were not included in the analysis due to delayed refrigeration. The limited sample size of this study needs to be addressed by larger studies even though many previous investigators have examined comparable sample size [11], [12]. The strength of our study, however, lies in the ethnically homogeneous nature of population which was enrolled from a single largest tertiary care centre in the region catering to over 1,50,000 general patients annually.

### Inclusion and Exclusion Criteria

The inclusion criteria for patients in both groups included those with age 50 years or older with the diagnosis of AMD. AMD was defined by geographic atrophy and/or choroidal neovascularization with drusen more than five in at least one eye. The controls constituting the study included those that were of age 50 years or older and had no drusen or no more than 5 drusen with absence of other diagnostic criteria for AMD.

The exclusion criteria included the retinal diseases involving the photoreceptors and/or outer retinal layers other than AMD loss such as high myopia, retinal dystrophies, central serious retinopathy, vein occlusion, diabetic retinopathy, uveitis or similar outer retinal diseases that have been present prior to the age of 50 and opacities of the ocular media, limitations of papillary dilation or other problems sufficient to preclude adequate stereo fundus photography. These conditions include occluded pupils due to synechiae, cataracts and opacities due to ocular diseases.

### Diagnosis of AMD

A retina specialist diagnosed all patients by ophthalmologic examination for best corrected visual acuity, slit lamp biomicroscopy of anterior segment and dilated fundus examination. All AMD patients were subjected to optical coherence tomography (OCT) and fluorescein fundus angiography (FFA). The diagnosis of AMD was based on FFA and ophthalmoscopic findings.

### Demographic Information

The demographic details were obtained by a trained interviewer using a standardized risk factor questionnaire. A written informed consent form signed by each participant, which included the written risk factor questionnaire was taken from each participant. The details such as age, sex, race, smoking etc as self reported by participants were entered in the data base for analysis. Smokers were defined as having smoked at least 1 cigarette per day for at least 6 months and divided into smokers and never smokers. Comorbidity was determined based on the participant’s responses to whether a physician had ever told them for diagnosis of any major neurological, metabolic or cardiovascular illness.

### Selection of Single-nucleotide Polymorphisms

The selected single-nucleotide polymorphisms (SNPs) in our study were either previously studied in other ethnic populations for association with AMD or other inflammatory diseases and chosen due to their reputed functional significance. The details are enumerated in Table 1.

**Table 1:** Description of SNPs genotyped.

Gene (RefSeq)	SNP	Chromosome position	Position in reference to 5′ UTR	Amino acid translation	Minor Allele
**CCL2 (NM_0029823)**	rs4586	17q11.2	+T974C	Cys35Cys	C
**CCR2 (NM_0006482)**	rs1799865	3p21.31	+T4439C	Asn260Asn	C

### Serum, PBMCs and DNA Isolation

About 8.0 ml of blood sample was collected from all subjects. About 3.0 ml of blood sample was left for 1 hour at 37°C and allowed to clot. Serum was subsequently separated in serum separator tube (BD Biosciences, USA) after centrifugation at 3000 rpm for 30 minutes. From rest of the blood PBMCs were isolated as per Histopaque-1077 (Sigma, USA) instruction sheet provided by the vendor. Briefly, 5.0 ml blood was layered on equal volume of Histopaque-1077 followed by centrifugation at 1800 rpm for 30.0 mins at room temperature. PBMCs were collected from plasma/Histopaque-1077 interface. Aliquots of PBMCs were stored in 90% fetal bovine serum (FBS, HiMedia, India) + 10% dimethyl sulphoxide (DMSO, Sigma, USA) and kept at −80°C until flow cytometry was done. Genomic DNA was extracted from PBMCs using a commercially available genomic DNA extraction and purification kit (INVITROGEN and QIAGEN) according to the manufacturer’s protocol. The samples were labeled, coded and stored.

**Table 2:** Demographic characteristics of Controls and AMD patients.

Variables	AMD	Controls
Total	133	80
Wet AMD	95 (71.4%)	–
Dry AMD	38 (28.6%)	–
Avastin treated	68	
Not treated with Avastin	27	
Duration of disease¥	23 ± 2.6 (M)	
Age†	66.56 ± 7.6	54.24±7.01
Male	88 (66.2%)	57 (71.2%)
Female	45 (33.8%)	23 (28.7%)

Clinical and demographic details of subjects. AMD, age related macular degeneration; M, Months; Age, Age of onset; Values are mean ± SD or (percentage),

† Unpaired, independent 2-tailed student t test analysis showed that mean age differ significantly among the groups (p = 0.02),

¥ Duration of disease is the interval between appearance of first symptom of AMD and collection of sample. AMD subjects were asked to provide all clinical and demographic details at the age of disease-onset.

### Real Time PCR

SNP (Single neucleotide polymorphism) was analyzed by using real time PCR, and was performed in the 48 wells model Step One^TM^ (Applied Biosystems Inc., Foster city, CA) using published TaqMan® SNP Genotyping Assays. Real time PCR was carried out for 20.0 µl containing 10 ul master mix, 5 ul Assay (Applied Biosystems), 20 ng DNA and molecular biology grade water was added to make the volume 20.0 µl. All reactions were carried out using TaqMan® SNP Genotyping Assays (Applied Biosystems) according to manufacturer’s recommendations. Two reporter dyes VIC and FAM were used to label the Allele 1 and 2 probes and a 5′ Nuclease Assay was carried out. Negative controls included the PCR mix without DNA. Software StepOneTM v 2.0 (Applied Biosystems Inc., Foster city, CA) was used to perform amplification and to estimate SNP. After PCR amplification the Sequence Detection System (SDS) Software was used to import the fluorescence measurements made during the plate read to plot fluorescence (Rn) values.

**Figure 1:**
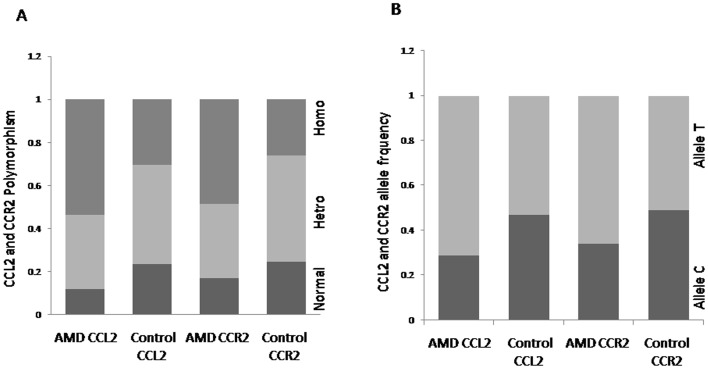
A) Genotype distribution (y-axis) of CCL2 and CCR2 polymorphism in the AMD patients compared to the control group (x-axis) in percentages. B) Allele frequency (y-axis) of CCL2 and CCR2 polymorphism in the AMD patients compared to the control group (x-axis) in percentages.

**Figure 2:**
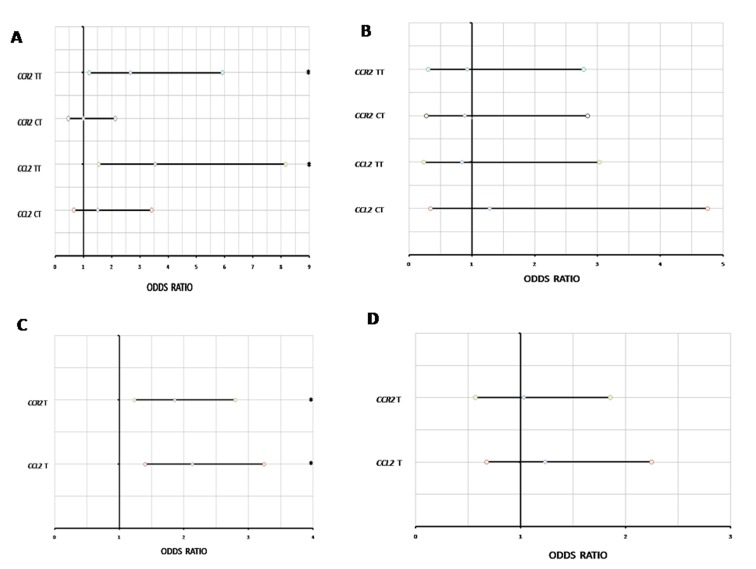
A) Univariate logistic regression analysis in AMD patients with CCL2 and CCR2 polymorphisms as independent and normal controls as a dependent variable. B) Univariate logistic regression analysis in Wet AMD patients with CCL2 and CCR2 polymorphisms as independent and Dry AMD as a dependent variable. C) Univariate logistic regression analysis in AMD patients with CCL2 and CCR2 alleles frequency as independent and normal controls as a dependent variable. D) Univariate logistic regression analysis in Wet AMD patients with CCL2 and CCR2 alleles frequency as independent and Dry AMD as a dependent variable. *p,0.05.

### Total Protein Estimation

Total protein was estimated using Bradford assay. The estimation of total protein was performed according to manufacturer’s recommendations. Briefly, serum samples were diluted 1500 times in double distilled water. Bovine Serum Albumin (BSA) served as the standard. Diluted samples and BSA standard protein were mixed with coomassie brilliant blue G–250 dye (Bradford reagent) in 4∶1 ratio followed by incubation at room temperature for 10–15 minutes. The absorbance was read at 595 nm in Microplate reader (680XR Biorad, Hercules, CA, USA). The standard curve of BSA was estimated with linear or quadratic fit models.

**Table 3:** Effect of CCL2 rs4586 and CCR2 rs1799865 variants on disease phenotype.

			Unadjusted p value			Multivariate analysis, adjusted for age			Multivariate analysis, adjusted for gender		
Genotype	Number (frequency)		OR	95%CI	p-Value	OR	95%CI	p- Value	OR	95%CI	p- Value
**CCL2 rs4586**											
	**AMD**	**Controls**									
**CC**	15 (0.118)	18 (0.236)	Reference			Reference			Reference		
**CT**	44 (0.346)	35 (0.461)	1.509	0.667–3.413	0.324	0.950	0.227–3.980	0.944	1.523	0.665–3.486	0.320
**TT**	68 (0.536)	23 (0.303)	3.548	1.543–8.157	0.003	0.517	0.107–2.494	0.411	0.300	0.129–0.695	0.005
	**Wet AMD**	**Dry AMD**									
**CC**	11(0.118)	4 (0.118)	Reference			Reference			Reference		
**CT**	30 (0.323)	14 (0.412)	1.283	0.347–4.749	0.709	2.450	0.388–15.46	0.340	1.254	0.335–4.686	0.737
**TT**	52 (0.559)	16 (0.471)	0.846	0.237–3.026	0.797	0.334	0.051–2.191	0.253	1.338	0.364–4.915	0.661
**CCR2 rs1799865**											
	**AMD**	**Controls**									
**CC**	22 (0.172)	19 (0.246)	Reference								
**CT**	44 (0.344)	38 (0.494)	1.00	0.472–2.121	1.00	2.147	0.558–8.232	0.267	1.00	0.472–2.212	0.999
**TT**	62 (0.484)	20 (0.260)	2.677	1.210–5.924	0.015	0.126	0.023–0.679	0.016	0.379	0.171–0.840	0.017
	**Wet AMD**	**Dry AMD**									
**CC**	16 (0.168)	6 (0.182)	Reference								
**CT**	33 (0.347)	11 (0.333)	0.889	0.279–2.836	0.842	0.404	0.072–2.249	0.301	0.875	0.275–2.789	0.822
**TT**	46 (0.484)	16 (0.485)	0.928	0.310–2.779	0.893	1.058	0.210–5.330	0.945	1.108	0.376–3.261	0.853

This table summarizes the genotype frequencies for the single-nucleotide polymorphisms (SNPs) in CCL2 rs4586 and CCR2 rs1799865 among patients with age-related macular degeneration (AMD) and control subjects. Genotype distributions were in Hardy-Weinberg equilibrium. The p-value represents comparison of risk significance between AMD cases and controls. OR indicates odds ratio and CI refers to confidence interval.

**Table 4:** Allele frequency of CCL2 and CCR2 in AMD and Normal controls.

Allele	Number (frequency)		OR	95%CI	p- Value
**CCL2 rs4586**					
	**AMD**	**Controls**			
**C**	74 (0.29)	71 (0.47)	**Reference**		
**T**	180 (0.71)	81 (0.53)	2.132	1.403–3.238	0.0003
					
	Wet AMD	Dry AMD			
**C**	52 (0.28)	22 (0.32)	**Reference**		
**T**	134 (0.72)	46 (0.68)	1.232	0.676–2.246	0.49
**CCR2 rs1799865**					
	**AMD**	**Controls**			
**C**	88 (0.34)	76 (0.49)	**Reference**		
**T**	168 (0.66)	78 (0.51)	1.86	1.237–2.796	0.002
					
	Wet AMD	Dry AMD			
**C**	65 (0.34)	23 (0.35)	**Reference**		
**T**	125 (0.66)	43 (0.65)	1.028	0.571–1.852	0.92

This table summarizes the allele frequencies for the single-nucleotide polymorphisms (SNPs) in CCL2 rs4586 and CCR2 rs1799865 among patients with age-related macular degeneration (AMD) and control subjects. The p-value represents comparison of risk significance between AMD cases and controls. OR indicates odds ratio and CI refers to confidence interval.

### Enzyme Linked Immunosorbant Assay (ELISA)

The expression of CCL2 was analyzed using commercially available enzyme linked immunosorbant assay (RayBio, Norcross, Cat#: ELH-MCP1-001) as per manufacturer’s protocol and absorbance was read at 450 nm in Microplate reader (Biorad 680XR, Hercules, CA, USA). Sample assays were performed in duplicate. This assay recognizes recombinant human CCL2 with minimum detectable dose of CCL2 typically less than 2 pg/ml. The standard curve was plotted using linear model and results were obtained after normalization with total protein.

**Figure 3:**
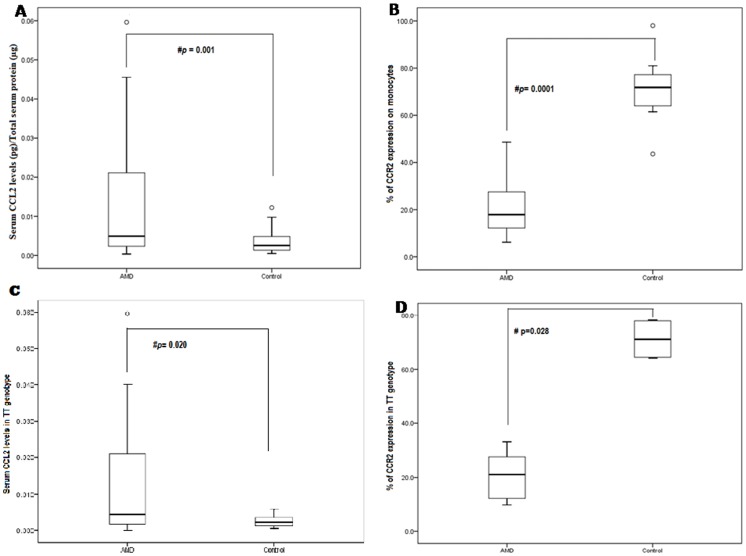
A) Serum levels of CCL2 in AMD and normal controls. B) Percentage (%) of PBMCs expressing CCR2 protein in AMD patients and Normal controls. C) Serum levels of CCL2 in TT genotype of AMD and normal controls. D) Percentage (%) of PBMCs expressing CCR2 protein in TT genotype of AMD patients and Normal controls. Boxes include values from first quartile (25th percentile) to third quartile (75th percentile). Lower and upper error bar refers to 10th and 90th percentile respectively. The thick horizontal line in the box represents median for each dataset. Outliers and extreme values are shown in circles and asterisk respectively. Levels of CCL2 were normalized to total protein. # indicates significant difference (p < 0.05) between the given conditions. Data was analyzed by Mann Whitney U Test. AMD, Age Related Macular Degeneration; CCL2, Chemokine ligand 2; CCR2, Chemokine Receptor 2; pg, picogram; µg, microgram.

**Figure 4:**
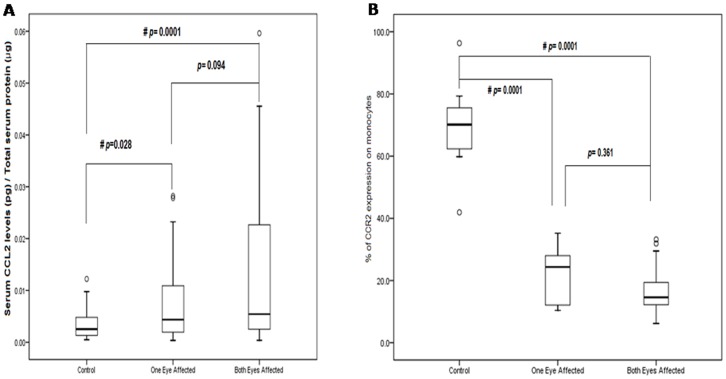
**A) Serum levels of CCL2 in normal controls, AMD patients affected in one eye and AMD patients affected in both eyes.** B) Percentage (%) of PBMCs expressing CCR2 protein in normal controls, AMD patients affected in one eye and AMD patients affected in both eyes. Boxes include values from first quartile (25th percentile) to third quartile (75th percentile). Lower and upper error bar refers to 10th and 90th percentile respectively. The thick horizontal line in the box represents median for each dataset. Outliers and extreme values are shown in circles and asterisk respectively. Levels of CCR2 were normalized to total protein. # indicates significant difference (p < 0.05) between the given conditions. Data was analyzed by Mann Whitney U Test. CCL2, Chemokine ligand 2; CCR2, Chemokine Receptor 2; pg, picogram; µg, microgram.

### Flow Cytometry

Flow cytometry was used to study the expression levels of surface receptors namely hCCR2 in PBMCs of normal subjects and AMD patients. ∼3×10^5^ PBMCs were initially processed for blocking with Fc blocker (1.0 µg, purified human IgG, R&D Systems Inc., Minneapolis, MN, USA) for 15 mins at room temperature with 0.2 ml of 0.1% sodium azide (Sigma, Germany) in 1× Ca^2+^ and Mg^2+^ free phosphate buffer saline (PBS) (HiMEDIA, India, pH = 7.2–7.4). Cell suspension was then incubated with primary labeled anti-hCCR2 - Allophycocyanin (0.1 µg, R&D Systems Inc., Minneapolis, MN, USA) antibody for 45 mins on ice in dark in 0.2 ml of fluorescence-activated cell sorter (FACS) buffer. Labeled antibody incubation was followed by two washings with 1× PBS at 5,000 rpm for 5 mins at 4°C. Finally, the cells were reconstituted in 250.0 µl of 1X PBS and analyzed in flow cytometer. Approximately 10,000 viable PBMCs were gated based on their forward and side scatter profile, and acquired in each run. PBMCs gate was set to include both lymphocytes and monocytes where maximum CCR2 fluorescence was observed. Same gating was used between the experiments. Background signal was measured for each sample by acquiring unlabeled PBMCs as negative controls and normalized to the signal obtained from anit-hCCR2 labeled PBMCs. Acquired cells were then verified for expression of CCR2. All the analysis was done by acquisition of data within one hour of incubation on FACS CANTO (BD Biosciences, San Jose, CA) flow cytometer using FACS DIVA software (Becton Dickinson).

**Figure 5:**
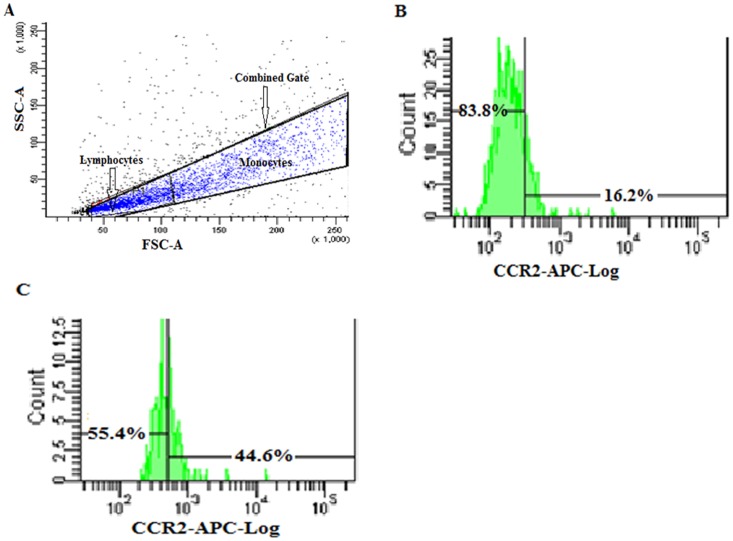
Percentage (%) of CCR2 + PBMCs in AMD patients and normal control subjects as measured by Flow Cytometry. (A) Dot plot showing side and forward scatter analysis of purified unlabeled PBMCs (large combined gate) from a AMD patient. PBMCs consists of two distinct populations namely lymphocytes and monocytes. Approximate lymphocytes and monocytes populations are indicated as smaller gates. Events outside the PBMCs gate represent cell debris and granulocytes. Same gating has been used for PBMCs from each AMD and normal control sample. ∼10,000 events have been acquired in each experiment. X-axis represents population cell size in forward scatter (FSC) and y-axis represents population cell granularity in side scatter (SSC). (B,C) Single parameter representative histogram of flow cytometric expression pattern of CCR2 on gated PBMCs is showing decreased number of CCR2 expressing PBMCs in AMD (16.2%; B) as compared to normal control (44.6%; C). Number of cells is represented along y-axis and blue APC fluorescence along x-axis. Appropriate unlabeled PBMCs were used to set marker in histogram and measure background fluorescence. APC, allophycocyanin; CCR2, chemokine receptor 2; PBMCs, peripheral blood mononuclear cells; AMD, Age related macular degeneration.

### Statistical Analysis

In order to see whether the data is normally distributed, Normal-quantile (Q-Q) plots were constructed. After establishing the normality for wet AMD cases, a parametric one-way analysis of variance (ANOVA) followed by Fisher’s least significant difference (LSD) post-hoc test was applied to compare multiple groups. For comparison of two groups unpaired, student-t test with equal or unequal variance (Welch’s correction) was applied. For non-normal data, a non-parametric Kruskal-Wallis H test followed by Mann-Whitney-U test was applied. The real time PCR estimated genotypes for each mutation were stratified for heterozygosity, and homozygosity for the respective allelic variant. Pearson’s Chi-square test was applied to study the association between various groups. Genotype distributions were analyzed by logistic regression, integrating adjustments for age and gender. Genotypic associations and odds ratios (ORs) with 95% confidence intervals (CI) were estimated by binary logistic regression. The *p* ≤0.05 was considered to be significant. Statistical analysis was performed with the help of SPSS 16.0 software.

## Results

Summary statistics of all-important variables have been obtained and reported in Table 2.

### rs4586 and rs1799865 Polymorphism in AMD Patients

To analyze the spectrum of polymorphism in CCL2 and CCR2 gene, real time PCR was used. The genotypes were in Hardy-Weinberg equilibrium. Genotype and allele frequencies of the polymorphisms of the genes CCL2 and CCR2 have been listed in the Table 3, 4 and Figure 1. The genotype and allele frequency for both CCL2 and CCR2 was found to be significantly different between AMD and normal controls. The TT genotype was more frequent in AMD patients than in controls for both CCL2 and CCR2 (OR = 3.548, p = 0.003, CI = 1.543–8.157 and OR = 2.677, p = 0.015, CI = 1.210–5.924, respectively, Table 3; Figure 2A). The study showed that the TT risk variant of CCL2 and CCR2 is associated with AMD (Figure 2A). The individuals having CT genotype in CCL2 and CCR2 revealed no risk of developing AMD (Figure 2A). Logistic regression analysis for food habits, existence of comorbidity and smoking habit revealed no significant difference between vegetarian/non-vegetarian, existence of comorbidity/without comorbidity and smokers/non-smokers AMD patients. However, when the comparison was done between AMD and controls, we found that TT genotype was more frequent among vegetarian AMD individuals than in vegetarian controls for CCL2 (OR = 5.574, p = 0.010, CI = 1.510–20.572, Table S1), TT genotype was more frequent in Non-vegetarian AMD than in Non-vegetarian controls for CCR2 (OR = 6.629, p = 0.008, CI = 1.652–26.59 Table S1) emphasizing the association of TT genotype in AMD. The AMD smokers and AMD never smokers showed significant TT frequency as compared to control smokers and control never smokers for CCL2 (OR = 5.80, p = 0.040, CI = 1.081–31.112 and OR = 3.380, p = 0.019, CI = 1.223–9.347, Table S2) and TT frequency was significantly higher in AMD smokers as compared to control smokers for CCR2 (OR = 15.6, p = 0.016, CI = 1.662–146.4, Table S2). However, there was no significant difference on the basis of comorbidity for CCL2 and CCR2 genotypes (Table S3). The frequency of allele T in CCL2 (rs4586) was found to be significantly higher in AMD patients (0.71%) as compared to the controls (0.53%) (OR = 2.132, p = 0.0003, CI = 1.403–3.238, Table-4, Figure 2C). CCR2 (rs1799865) allele frequency of allele T was also significantly higher in AMD patients (0.66%) as compared to the controls (0.51%) (OR = 1.86, p = 0.002, CI = 1.237–2.792, Table 4, Figure 2C). We did not find any significant difference in genotype and allele frequency between wet and dry AMD patients (Table 3&4; Figure 2B&D). The difference was also not significant when compared between wet AMD patients ie minimally classic, predominantly classic and occult (data not shown). There was no significant difference when compared between those wet variant of AMD patients who received Avastin treatment (dose 1.25 mg in 0.05 ml) and those that did not (data not shown).

### Multiple Logistic Regression Analysis

To analyze the association of genetic polymorphism and other risk factors with AMD simultaneously, we performed unconditional logistic regression analysis and obtained optimized model. We analyzed both age and gender as risk factors which have been shown to be associated with AMD previously. The Hosmer-Lemenshow test shows that the data fits well to the logistic regression (p = 0.70). When multiple logistic regression analysis was carried out for age adjustment, we found that TT genotype showed significantly higher frequency for CCR2 rs1799865 in AMD as compared to controls (OR = 0.126, p = 0.016, and CI = 0.023–0.679, Table-3) and multiple logistic regression adjustment analysis for gender showed that TT genotype was at significantly higher frequency for CCL2 rs4586 and CCR2 rs1799865 for AMD patients (Table-3). Gender adjustment also showed significant difference in genotype TT for Vegetarian AMD, never smokers AMD (CCL2 rs4586) and comorbidity and smoker AMD (CCR2 rs1799865 Table S1, S2, S3).

### Decreased CCR2 and Increased CCL2 Levels

ELISA estimation revealed elevated levels of serum CCL2 in AMD patients as compared to normal controls (Figure 3 A; p = 0.001). No difference was observed in CCL2 levels for wet and dry AMD (p = 0.327). CCL2 concentration was significantly elevated in the patients affected in one or both eyes with AMD as compare to controls (Figure 4A). However, flow cytometry analysis of PBMCs of AMD patients and normal controls indicates a significant decrease in proportion of CCR2 expressing PBMCs from AMD patients than those from normal controls (Figure 3B & 5; p = 0.0001). We found no significant difference in their expression between Dry and Wet AMD samples (p = 0.934). CCR2 expression was significantly lower in the patients affected in one eye or both eyes with AMD as compared to controls but the difference was not significant between one eye affected and both eyes affected (Figure 4B). The CCL2 ELISA and CCR2 FACS levels were not significant when compared between avastin treated & untreated wet AMD patients and between different classes of wet AMD i.e. minimally classic, predominantly classic and occult (data not shown). No association of cigarette smoking, alcohol and meat consumption with CCR2 and CCL2 levels in serum was observed upon univariate and multivariate analysis. The levels of CCL2 determined by ELISA and CCR2 expression estimated by FACS were corresponded to the TT polymorphism in CCL2 and CCR2 in between AMD and controls (Figure 3C&D).

## Discussion

The current study suggests that inflammation is essential part of the pathogenesis of AMD in the Indian AMD patients. After examining the involvement of gene polymorphism and levels of inflammatory genes with the risk of AMD, it is suggested that genetic variations in the genes encoding the inflammatory processes might confer susceptibility to AMD by altering the expression of these cytokines. The presence of risk genotype of these genes may increase the risk of AMD.

We examined the levels of CCL2, percentage of cells expressing CCR2 and two variants of these pro-inflammatory cytokine genes which have been studied for other ethnic populations for AMD [9] and shown to be linked with inflammatory diseases [13], [14] and were functional variants affecting expression or function of these genes. It must be mentioned that SNPs from CCL2 are previously known to affect CCL2 protein levels [15]. In acute inflammation expression of CCL2 in the retina and RPE increases [16]–[18], with oxiative stress in the RPE [19]. A recent study had shown that subretinal microglial cells (MCs) induce CCL5 and CCL2 in the RPE [20]. CCL2 mainly signals through CCR2 [21]. It has been shown that CCL2/ CCR2 signaling is involved in monocyte or microglial cells enrollment after laser injury [22]. Microglial cells or CCR2-expressing monocytes are present at some point in these models. In a clinical study Jonas et al showed that elevated intraocular levels of CCL2 are associated with exudative AMD [23] and in a mouse model of CNV [16]. CCL2 might therefore play a role in monocyte and MC recruitment to the subretinal space in AMD.

Besides our own work there are numerous reports using CCL2−/− or CCR2−/− mice in an attempt to translate the inflammatory mechanisms of AMD. Recently Chen et al has also shown that aged CCL2 or CCR2 deficient mice develop certain features of atrophic, but not angiogenic AMD-like changes, and represent an animal model for early stage human geographic atrophy [24]. Several studies have examined AMD susceptibility and analyzed SNPs from chemokine family. However, no evidence has been found for an association between common genetic variations of CCR2 and CCL2 with the etiology of AMD [9], [10] but this did not include North Indian patients. However, functional polymorphisms in these genes has been found to play a significant role in the development of other inflammatory diseases [13], [25], [26]. A family of structurally related chemotactic cytokines comprise chemokines that direct the migration of leukocytes throughout the body, both under pathological and physiological conditions [27]. CCR2 and CCL2 are key mediators in the infiltration of monocytes into foci of inflammation from blood. The CCL2 protein is expressed ubiquitously and exerts its effect after binding to its receptor CCR2 which leads to shape change, actin rearrangement and monocytes movement [28]. As CCL2 and CCR2 genes were considered as potential candidates genes in AMD animal model studies, we analyzed the evidence from genetic variation of CCL2 and CCR2 in human despite conflicting reports. The results of these finding support the postulation that mice deficient in these genes develop hallmarks of AMD [4] (i.e. lipofuscin, accumulation of drusen, photoreceptor atrophy, and CNV). The presence of AMD-like disease in these knockout mice had raised questions of whether CCR2 and CCL2 play a role in human AMD. On examining the two variants of these inflammatory cytokines it was found that these alleles and genotypes are in Hardy-Weinberg Equilibrium in AMD and control subjects. Earlier studies in animal models have shown that CCL2 and CCR2 are involved in the pathogenesis of AMD [4], [29], [30]. We have examined single polymorphism for CCL2 (rs4586) and CCR2 (rs1799865) with their levels for susceptibility of AMD. The CCL2 transcription may be influenced by the CCL2 (rs4586) SNP, which may act in association with the CCR2 (rs1799865) SNP, impacting the biological activity of the CCR2 receptor, and the CCL2/CCR2 messenger system.

Our study has revealed that the levels of CCL2 were higher and number of cells expressing CCR2 were lower in AMD patients as compared to controls which could be ascribed to the varying physiology of primates and rodents. This might be explained by proposing the activation of a negative feedback seeking to limit the inflammation caused by extravasations of activated monocytes/lymphocytes at the site of macular degeneration. We also found that the levels of CCL2 or percentage of cells expressing CCR2 did not significantly increase or decrease in the patients affected in one eye or those affected in both eyes. We are unable to rule out the local difference in CCL2 and CCR2 because we did not analyze the respective autopsies. The levels of CCL2 in TT genotype of rs4586 was significantly higher in AMD patients as compared to normal controls and the percentage of cells expressing CCR2 were significantly lower in TT genotype of rs1799865 in AMD patients as compared to normal controls which we are unable to explain. The risk of disease increases in individuals 2.6–3.5 times in those who present with genotype TT as compared to CC within both CCR2 (rs1799865) and CCL2 (rs4586) respectively. Individuals with T allele have higher risk of 1.8–2.1 times for developing AMD as compared to C allele for both CCR2 (rs1799865) and CCL2 (rs4586) respectively. We did not find any significant difference between food habit, comorbidity and smoking for AMD patients which indicates no association with disease.

To the best of our knowledge this is the first study suggesting synergy between the SNPs of CCL2 (rs4586) and its receptor CCR2 (rs1799865) with their protein levels in the development of AMD. Additional studies in larger populations comparing Asian and African and North Americans are needed to validation with larger sample size to allow for the confirmation or negation of an independent role of each of these SNPs on the risk of AMD development or verifying their mutual properties.

## Supporting Information

Table S1 **Logistic regression of the association CCL2, CCR2 and progression of AMD stratified by food habits.**
(DOC)

Table S2 **Logistic regression of the association CCL2, CCR2 and progression of AMD stratified by smoking.**
(DOC)

Table S3 **Logistic regression of the association CCL2, CCR2 and progression of AMD stratified by comorbidity.**
(DOC)
