# Supplementary material for: Single Nucleotide Polymorphisms in MCP-1 and Its Receptor Are Associated with the Risk of Age Related Macular Degeneration
Source: PLoS One. 2012 Nov 21;7(11):e49905. doi: 10.1371/journal.pone.0049905 (PMC3503775; doi:10.1371/journal.pone.0049905)
Supplement: Table S1 — Logistic regression of the association CCL2, CCR2 and progression of AMD stratified by food habits. (DOC) [file pone.0049905.s001.doc]

**Table S1**. Logistic regression of the association CCL2, CCR2 and progression of AMD stratified by food habits

|  |  | | **Unadjusted p value** | | | **Multivariate analysis, adjusted for age** | | | **Multivariate analysis, adjusted for gender** | | |
| --- | --- | --- | --- | --- | --- | --- | --- | --- | --- | --- | --- |
| **Genotype** | **Number (frequency)** | | **OR** | **95%CI** | **P-value** | **OR** | **95%CI** | **P-value** | **OR** | **95%CI** | **P-value** |
| **CCL2_4586** | | | | | |  |  |  |  |  |  |
|  | Vegetarian AMD | Vegetarian Controls |  |  |  |  |  |  |  |  |  |
| **CC** | 6 (.09) | 7 (0.19) | Reference |  |  |  |  |  |  |  |  |
| **CT** | 20 (0.28) | 20 (0.56) | 1.167 | O.333-4.089 | 0.810 | 2.00 | 0.174-23 | 0.578 | 1.205 | 0.34-4.27 | 0.773 |
| **TT** | 43 (0.62) | 9 (0.25) | 5.574 | 1.510-20.572 | 0.010 | 0.714 | 0.055-9.307 | 0.797 | 0.190 | 0.052-0.695 | 0.012 |
|  | Non Vegetarian AMD | Non Vegetarian Controls |  |  |  |  |  |  |  |  |  |
| **CC** | 9 (0.16) | 10 (0.31) | Reference |  |  |  |  |  |  |  |  |
| **CT** | 24 (0.41) | 12 (0.38) | 2.222 | 0.713-6.923 | 0.168 | 1.00 | 0.077-13.01 | 1.00 | 2.014 | 0.628-6.463 | 0.239 |
| **TT** | 25(0.43) | 10 (0.31) | 2.778 | 0.870-8.873 | 0.085 | 1.50 | 0.075-29.945 | 0.791 | 0.387 | 0.120-1.254 | 0.113 |
|  |  |  |  |  |  |  |  |  |  |  |  |
| **CCR2_1799865** | | | | | |  |  |  |  |  |  |
|  | Vegetarian AMD | Vegetarian Controls |  |  |  |  |  |  |  |  |  |
| **CC** | 17 (0.24) | 9 (0.26) | Reference |  |  |  |  |  |  |  |  |
| **CT** | 20 (0.29) | 14 (0.40) | 0.756 | 0.263-2.179 | 0.605 | 1.371 | 0.132-14.199 | 0.791 | 0.789 | 0.274-2.273 | 0.660 |
| **TT** | 33 (0.47) | 12 (0.34) | 1.456 | 0.513-4.134 | 0.481 | 0.228 | 0.042-1.234 | 0.086 | 0.708 | 0.249-2.013 | 0.517 |
|  | Non Vegetarian AMD | Non Vegetarian Controls |  |  |  |  |  |  |  |  |  |
| **CC** | 5 (0.09) | 8 (0.24) | Reference |  |  |  |  |  |  |  |  |
| **CT** | 24 (0.41) | 18 (0.55) | 2.133 | 0.597-7.624 | 0.244 | 2.500 | 0.236-26.480 | 0.447 | 2.096 | 0.579-7.582 | 0.259 |
| **TT** | 29 (0.50) | 7 (0.21) | 6.629 | 1.652-26.59 | 0.008 | * | * | * | 0.144 | 0.035-0.591 | 0.144 |

* **The value could not be complied because of the equal frequencies.** **This table summarizes the genotype frequencies for the single-nucleotide polymorphisms (SNPs) in CCL2 rs4586 and CCR2 rs1799865 among food habit of patients with age-related macular degeneration (AMD) and control subjects. Genotype distributions were in Hardy-Weinberg equilibrium. The p-value represents comparison of risk significance between AMD cases and controls. OR indicates odds ratio and CI refers to confidence interval.**
