# Supplementary material for: Single Nucleotide Polymorphisms in MCP-1 and Its Receptor Are Associated with the Risk of Age Related Macular Degeneration
Source: PLoS One. 2012 Nov 21;7(11):e49905. doi: 10.1371/journal.pone.0049905 (PMC3503775; doi:10.1371/journal.pone.0049905)
Supplement: Table S2 — Logistic regression of the association CCL2, CCR2 and progression of AMD stratified by smoking. (DOC) [file pone.0049905.s002.doc]

**Table S2. Logistic regression of the association CCL2,** CCR2 and progression of AMD stratified by smoking

|  |  | | **Unadjusted p value** | | | **Multivariate analysis, adjusted for age** | | | **Multivariate analysis, adjusted for gender** | | |
| --- | --- | --- | --- | --- | --- | --- | --- | --- | --- | --- | --- |
| **Genotype** | **Number (frequency)** | | **OR** | **95%CI** | **P value** | **OR** | **95%CI** | **P value** | **OR** | **95%CI** | **P value** |
| **CCL2_4586** | | | | | |  |  |  |  |  |  |
|  | AMD Smokers | Control Smokers |  |  |  |  |  |  |  |  |  |
| **CC** | 5 (0.09) | 4 (0.33) | Reference |  |  |  |  |  |  |  |  |
| **CT** | 22 (0.39) | 4 (0.33) | 4.400 | 0.810-23.899 | 0.086 | * | * | * | 5.333 | 0.891-31.91 | 0.067 |
| **TT** | 29 (0.52) | 4 (0.33) | 5.800 | 1.081-31.112 | 0.040 | * | * | * | 0.200 | 0.037-1.08 | 0.061 |
|  | AMD Non Smokers | Control Non Smokers |  |  |  |  |  |  |  |  |  |
| **CC** | 10 (0.14) | 13 (0.23) | Reference |  |  |  |  |  |  |  |  |
| **CT** | 22 (0.31) | 28 (0.50) | 1.021 | 0.377-2.765 | 0.967 | 1.389 | 0.131-14.745 | 0.785 | 0.877 | 0.314-2.455 | 0.803 |
| **TT** | 39 (0.55) | 15 (0.27) | 3.380 | 1.223-9.347 | 0.019 | 0.233 | 0.025-2.153 | 0.199 | 0.339 | 0.120-0.955 | 0.041 |
| **CCR2_1799865** | | | | | |  |  |  |  |  |  |
|  | AMD Smokers | Control Smokers |  |  |  |  |  |  |  |  |  |
| **CC** | 10 (0.18) | 6 (0.50) | Reference |  |  |  |  |  |  |  |  |
| **CT** | 21 (0.37) | 5 (0.42) | 2.520 | 0.618-10.276 | 0.197 | * | * | * | 2.700 | 0.613-11.89 | 0.189 |
| **TT** | 26 (0.46) | 1 (0.08) | 15.600 | 1.662-16.405 | 0.016 | * | * | * | 0.072 | 0.008-0.683 | 0.022 |
|  | AMD Non Smokers | Control Non Smokers |  |  |  |  |  |  |  |  |  |
| **CC** | 12 (0.17) | 11 (0.20) | Reference |  |  |  |  |  |  |  |  |
| **CT** | 23 (0.33) | 27 (0.48) | 0.781 | 0.290-2.100 | 0.624 | 5.429 | 0.501-58.85 | 0.164 | 0.822 | 0.302-2.238 | 0.702 |
| **TT** | 36 (0.50) | 18 (0.32) | 1.833 | 0.678-4.957 | 0.232 | 0.143 | 0.011-1.859 | 0.137 | 0.531 | 0.191-1.474 | 0.224 |

* **The value could not be complied because of the equal frequencies. This table summarizes the genotype frequencies for the single-nucleotide polymorphisms (SNPs) in CCL2 rs4586 and CCR2 rs1799865 among smoking habit of patients with age-related macular degeneration (AMD) and control subjects. Genotype distributions were in Hardy-Weinberg equilibrium. The p-value represents comparison of risk significance between AMD cases and controls. OR indicates odds ratio and CI refers to confidence interval.**
