# Supplementary material for: Single Nucleotide Polymorphisms in MCP-1 and Its Receptor Are Associated with the Risk of Age Related Macular Degeneration
Source: PLoS One. 2012 Nov 21;7(11):e49905. doi: 10.1371/journal.pone.0049905 (PMC3503775; doi:10.1371/journal.pone.0049905)
Supplement: Table S3 — Logistic regression of the association CCL2, CCR2 and progression of AMD stratified by comorbidity. (DOC) [file pone.0049905.s003.doc]

|  |  | | **Unadjusted p value** | | | **Multivariate analysis, adjusted for age** | | | **Multivariate analysis, adjusted for gender** | | |
| --- | --- | --- | --- | --- | --- | --- | --- | --- | --- | --- | --- |
| **Geno-type** | **Number (frequency)** | | **OR** | **95%CI** | **P Value** | **OR** | **95%CI** | **P-value** | **OR** | **95%CI** | **P-value** |
| **CCL2_4586** | | | | | |  |  |  |  |  |  |
|  | AMD with Comorbidity | Controls with Comorbidity |  |  |  |  |  |  |  |  |  |
| **CC** | 6 (0.07) | 2 (0.16) | Reference |  |  |  |  |  |  |  |  |
| **CT** | 31 (0.36) | 8 (0.68) | 1.292 | 0.218-7.652 | 0.778 | 1.333 | 0.105-17.00 | 0.825 | 1.209 | 0.191-7.644 | 0.840 |
| **TT** | 50 (0.57) | 2 (0.16) | 8.333 | 0.985-70.48 | 0.052 | * | * | * | 0.125 | 0.014-1.096 | 0.060 |
|  | AMD without Comorbidity | Controls without Comorbidity |  |  |  |  |  |  |  |  |  |
| **CC** | 9 (0.23) | 15 (0.28) | Reference |  |  |  |  |  |  |  |  |
| **CT** | 12 (0.31) | 22 (0.41) | 0.909 | 0.307-2.691 | 0.863 | 1.200 | 0.154-9.355 | 0.862 | 1.035 | 0.343-3.121 | 0.951 |
| **TT** | 18 (0.46) | 17 (0.31) | 1.765 | 0.612-5.090 | 0.293 | 1.167 | 0.175-7.757 | 0.873 | 0.661 | 0.219-1.990 | 0.462 |
| **CCR2_1799865** | | | | | |  |  |  |  |  |  |
|  | AMD with Comorbidity | Controls with Comorbidity |  |  |  |  |  |  |  |  |  |
| **CC** | 16 (0.18) | 5 (0.42) | Reference |  |  |  |  |  |  |  |  |
| **CT** | 29 (0.34) | 4 (0.33) | 2.266 | 0.532-9.654 | 0.269 | 2.00 | 0.173-23.176 | 0.579 | 2.143 | 0.487-9.429 | 0.313 |
| **TT** | 42 (0.48) | 3 (0.25) | 4.375 | 0.935-20.46 | 0.061 | * | * | * | 0.203 | 0.041-0.991 | 0.049 |
|  | AMD without Comorbidity | Controls without Comorbidity |  |  |  |  |  |  |  |  |  |
| **CC** | 6 (0.15) | 12 (0.22) | Reference |  |  |  |  |  |  |  |  |
| **CT** | 14 (0.36) | 26 (0.48) | 1.077 | 0.332-3.490 | 0.902 | 3.250 | 0.283-37.258 | 0.344 | 1.067 | 0.335-3.399 | 0.913 |
| **TT** | 19 (0.49) | 16 (0.30) | 2.375 | 0.727-7.763 | 0.152 | 0.462 | 0.041-5.158 | 0.530 | 0.436 | 0.134-1.412 | 0.166 |

**Table S3. Logistic regression of the association CCL2, CCR2 and progression of AMD stratified by comorbidity**

*** The value could not be complied because of the equal frequencies. This table summarizes the genotype frequencies for the single-nucleotide polymorphisms (SNPs) in CCL2 rs4586 and CCR2 rs1799865 among comorbity status of patients with age-related macular degeneration (AMD) and control subjects. Genotype distributions were in Hardy-Weinberg equilibrium. The p-value represents comparison of risk significance between AMD cases and controls. OR indicates odds ratio and CI refers to confidence interval**
